# Transcriptional induction of the IMD signaling pathway and associated antibacterial activity in the digestive tract of cat fleas (*Ctenocephalides felis*)

**DOI:** 10.1186/s13071-024-06613-x

**Published:** 2024-12-30

**Authors:** Katie Weber, Dhruva Karnik, Lisa D. Brown

**Affiliations:** Department of Biology, Georgia Southern University, 4324 Old Register Rd., Statesboro, GA 30460 USA

**Keywords:** Antimicrobial peptides, *Bartonella*, Blood-feeding, Insect immunity, Pulicidae, Siphonaptera

## Abstract

**Background:**

Fleas are insect vectors that transmit several Gram-negative bacterial pathogens acquired by ingesting infected vertebrate blood. To combat foodborne illness, insect midgut epithelial cells are armed with efficient microbial recognition and control systems, such as the immune deficiency (IMD) pathway that regulates the expression of antimicrobial peptides (AMPs). However, despite their medical and veterinary importance, relatively little is known about the IMD signaling pathway and production of AMPs in the digestive tract of cat fleas (*Ctenocephalides felis*).

**Methods:**

In the present study, we measured the expression of target genes comprising the IMD pathway, as well as corresponding AMP transcripts, in the digestive tract of *C. felis* following exposure to three different species of bacteria: Gram-negative *Bartonella henselae* (a flea-borne pathogen), Gram-negative *Serratia marcescens* (a model laboratory species), and Gram-positive *Micrococcus luteus* (a model laboratory species). Additionally, we examined the antibacterial activity of proteins isolated from the flea digestive tract in vitro following bacterial challenge and at different days post adult emergence to determine if feeding-induced antibacterial activity varies with age.

**Results:**

In our analysis of *C. felis*, we observed an increase in the expression of representative IMD pathway genes and associated AMP transcripts, indicating the activation of the IMD pathway. Furthermore, our results revealed that different bacterial species elicit distinct transcriptional profiles of IMD pathway genes, suggesting a species-specific response to bacterial invasion. We found that the gut of *C. felis* produces antibacterial molecules as a localized defense mechanism. Additionally, we observed that proteins with antimicrobial properties are synthesized as part of local defense mechanisms in the gut, with differential patterns of antibacterial activity related to infection status and age.

**Conclusions:**

Our findings provide essential insights into the potential mechanisms by which cat fleas regulate immune responses in their digestive tract against different bacterial species.

**Graphical Abstract:**

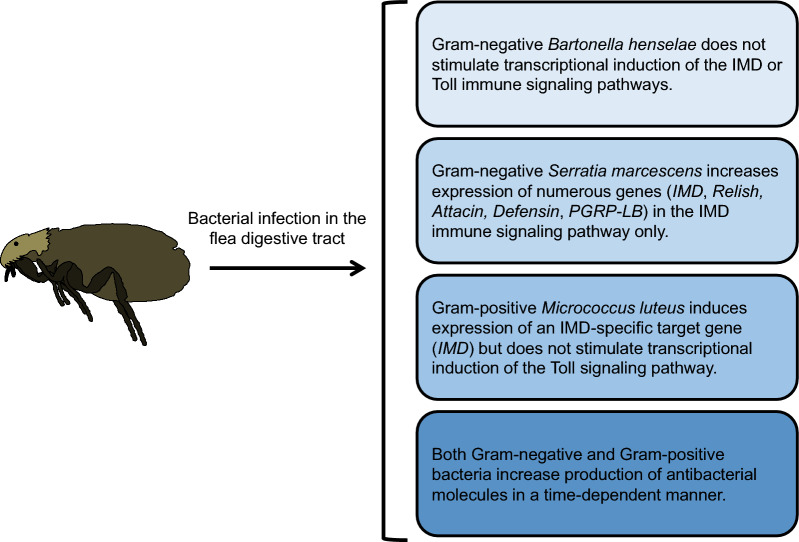

**Supplementary Information:**

The online version contains supplementary material available at 10.1186/s13071-024-06613-x.

## Background

The insect digestive tract is one of the most vulnerable sites to microbial intruders [1, 2]. Through the ingestion of contaminated food, the intestinal epithelium provides pathogens access to colonize and invade host tissues. To prevent contact between surface epithelia and infectious agents, a protective chitinous membrane (peritrophic matrix) is formed around food in the gut of most insects [3]. Additionally, midgut epithelial cells are armed with efficient systems for pathogen recognition and control: the Duox system that produces reactive oxygen species (ROS), and the immune deficiency (IMD) pathway that regulates the expression of antimicrobial peptides (AMPs) [2]. The secretion of AMPs in the gut lumen is a significant component of the antibacterial response in insects, especially against Gram-negative bacterial pathogens [4].

The relationship between ingesting bacterial pathogens and activation of the IMD pathway is best understood in the fruit fly (*Drosophila melanogaster*) gut [5–9]. First, the transmembrane receptor peptidoglycan recognition protein LC (PGRP-LC) recognizes peptidoglycans released by replicating Gram-negative bacteria, and recruits the adaptor protein IMD. Next, IMD interacts with the adaptor protein FAS-associated death domain (FADD) and binds to the apical caspase Dredd, after which cleavage, phosphorylation, and nuclear translocation of the transcription factor Relish occurs. The nuclear factor kappa-light-chain-enhancer of activated B cells (NF-κB) domain of Relish initiates the transcription of several insect AMP genes, such as *attacin*, *cecropin*, *defensin*, *diptericin*, *drosocin*, and *drosomycin*, which have a wide range of inhibitory effects against bacterial pathogens [4]. Finally, peptidoglycan recognition protein LB (PGRP-LB) is released into the gut lumen, degrading residual peptidoglycan into nonimmunogenic fragments and negatively regulating the IMD pathway. The IMD pathway also plays a role in the *Drosophila* gut defense against Gram-positive bacteria, as the Toll pathway—a key activator of the NF-κB-like proteins Dif and Dorsal upon recognition of Gram-positive peptidoglycan—is considered nonfunctional in the fly intestine [10]. In this case, the IMD pathway is necessary for expelling ingested bacteria through defecation instead of producing AMPs.

Cat fleas (*Ctenocephalides felis*) are insect vectors that transmit several Gram-negative bacterial pathogens, including *Bartonella henselae*, *Rickettsia felis*, *Rickettsia typhi*, and *Yersinia pestis* [11]. Adult fleas primarily acquire these infections by blood-feeding on a bacteremic vertebrate host. Two unique features of the cat flea digestive tract differ from other insects and blood-feeding arthropods. First, flea midgut epithelial cells do not produce a peritrophic matrix; thus, ingested microbes are not prevented from coming into direct contact with surface epithelia [12–14]. Second, cat fleas feed frequently and quickly process their bloodmeals to excrete host blood-proteins for the larvae to consume [15–17]. Therefore, it is hypothesized that flea-borne pathogens must bind to putative receptor proteins on flea midgut epithelial cells to avoid elimination. Once attached to the cell surface, obligate (*Rickettsia* spp.) and facultative (*Bartonella* spp.) intracellular pathogens can then induce their internalization by the host cell or aggregate to form a biofilm, as is postulated for *B. henselae* [18]. Consequently, the intestinal epithelium of cat fleas must rapidly produce a robust antimicrobial repertoire to fend off pathogen attachment and subsequent tissue invasion.

Despite their medical and veterinary importance, relatively little is known about the immune responses in the digestive tract of cat fleas, particularly regarding the induction of AMPs. Like other blood-feeding insects, an array of AMPs is suspected to compose the flea antibacterial response, but only *defensin* gene expression has been identified in *C. felis* [19, 20]. Moreover, the activation of immune signaling pathways upon exposure to different species of bacteria is poorly understood. The limited number of studies on *C. felis* immunity are primarily conducted using rickettsial pathogens, and the data indicate that the IMD pathway regulates these infections [21]. However, comparing immune activity against different types of bacteria (Gram-negative versus Gram-positive) and other species of flea-borne pathogens will provide valuable insights into the diverse range of potential immune responses exhibited by the cat flea vector. In the present study, we compared the transcriptional profiles of four representative IMD pathway genes (*PGRP-LC*, *IMD*, *Relish*, and *PGRP-LB*) from the digestive tract of *C. felis* following exposure to three different species of bacteria: Gram-negative *Bartonella henselae* (a flea-borne pathogen), Gram-negative *Serratia marcescens* (a model laboratory species), and Gram-positive *Micrococcus luteus* (a model laboratory species). We also measured the relative transcript abundance of two AMPs (*attacin* and *defensin*) frequently associated with the IMD pathway and three representative Toll pathway genes (*PGRP-SA*, *Toll*, and *cactus*) to determine their activity, if any, in response to bacterial infection. Lastly, we examined the antibacterial activity of proteins isolated from the flea digestive tract in vitro following bacterial challenge and at different days post adult emergence to determine if feeding-induced antibacterial activity varies with age.

## Methods

### Flea source and maintenance

Adult cat fleas (*C. felis* (Bouché)), originally sourced from Elward II Laboratory (Soquel, CA, USA), were collected from a laboratory colony maintained at Georgia Southern University (GS) as previously described [22]. Starting in September 2023, as Elward II Laboratory is no longer operational, the GS colony was supplemented with adult cat fleas from Ecto Services, Inc. (Henderson, NC, USA). A portion of these new fleas were tested to verify the absence of *B. henselae* infection using quantitative real-time polymerase chain reaction (qPCR) analyses [23]. Adult fleas were given defibrinated bovine blood (Hemostat Laboratories, Dixon, CA, USA) using an artificial feeding system [24]. Male and female fleas were kept together during experimental treatments to maximize adult survival. However, only female fleas with visibly confirmed ingested blood were selected for further analyses. Unless stated otherwise, experimental treatment groups were created using blood-fed adult fleas between 3 and 5 days post-emergence.

### Bacterial cultures and generation of infected bloodmeals

During the course of this study, adult fleas were fed blood infected with one of the following bacterial species: Gram-negative *Bartonella henselae* (Regnery et al., Brenner et al. (ATCC 49882)), Gram-negative *Serratia marcescens* (D1, Carolina Biological Supply Company), and Gram-positive *Micrococcus luteus* (Carolina Biological Supply Company). *Bartonella henselae* was grown by inoculating 1 mL of tryptic soy broth (TSB) with 1 mL of a thawed vial of previously frozen primary stock culture. This suspension was added to either a commercially prepared tryptic soy agar (TSA) slant with 5% sheep blood from Carolina Biological Supply Company or a laboratory-prepared TSA slant with 5% bovine blood. These biphasic slants were incubated using one of two methods: (1) a candle extinction jar placed inside a shaking incubator at 37 °C and 200 rpm for approximately 3 days, or (2) a Ziploc^®^ bag with CO_2_ gas generators (Thermo Fisher Scientific™ AnaeroPack™ Gas Generator) inside a standard microbiological incubator at 37 °C for approximately 10 days. Before use in experiments, the broth pool of the biphasic slant was pelleted by centrifugation (15,000 × *g* for 3 min) and resuspended in fresh TSB. Both *S. marcescens* and *M. luteus* were grown overnight in a shaking incubator at 25 °C and 350 rpm in nutrient broth.

To create an infected bloodmeal, 200 μL of the bacterial culture was pelleted by centrifugation (15,000 × *g* for 5 min) and resuspended in 600 μL of heat-inactivated bovine blood (56 °C for 10 min). The infection dose was estimated beforehand by measuring the OD_600_ of the bacterial culture in a BioPhotometer D30 (Eppendorf, AG, Hamburg, Germany) and was standardized across all bacterial species (OD_600_ = 5). The absolute dose of *B. henselae* was determined by quantifying *Bartonella* gene copy numbers using qPCR and genomic DNA (gDNA) extracted from the bacterial culture (detailed below). In contrast, absolute doses for *S. marcescens* and *M. luteus* were determined by plating serial dilutions of the bacterial cultures on nutrient agar, growing them for 48 h at 25 °C, and counting the resultant colony forming units (CFUs). Across all experimental trials, the mean (± SEM) absolute dose of each bacterial species was as follows: (1) 1.96 × 10^10^ (± 6.09 × 10^9^) gene copies of *B. henselae*, (2) 8.58 × 10^7^ (± 3.14 × 10^7^) CFUs of *S. marcescens*, and (3) 3.16 × 10^7^ (± 6.17 × 10^6^) CFUs of *M. luteus*. Complete descriptive statistics of the absolute dose of each bacterial species are listed in Additional file 2: Table S2.

### Experimental treatment groups and measurement of bacterial infection

Adult fleas were placed into four groups and starved overnight before exposure to one of the following experimental treatments: (1) untreated blood (control), (2) blood infected with Gram-negative *B. henselae* (a flea-borne bacterial pathogen), (3) blood infected with Gram-negative *S. marcescens* (a model laboratory species), and (4) blood infected with Gram-positive *M. luteus* (a model laboratory species). Fleas were allowed to feed on the experimental treatment blood for either 4 h or 24 h (*n* = 8 groups total; four treatments at two time points). Immediately after each time point, a subset of fleas (*n* = 5) was removed to determine infection prevalence (proportion of fleas infected with bacteria) and infection intensity (mean number of bacteria in an infected flea) for each bacterial species. Specifically, each flea was placed in a 1.7 mL microcentrifuge tube, and tissues were pulverized using a sterile polypropylene micropestle. Akin to the quantification of *B. henselae* in culture, gDNA was extracted from individual flea samples and used to measure *Bartonella* gene copy numbers by qPCR (detailed below). For fleas infected with *S. marcescens* or *M. luteus*, 200 μL of phosphate-buffered saline (PBS) was added to the ground tissues, and a diluted sample for each flea was spread on nutrient agar plates. Plates were incubated at room temperature (25 °C) for 48 h, and the number of CFUs was counted.

### Quantification of *Bartonella henselae* by qPCR

Genomic DNA was extracted from bacterial cultures and individual fleas using the PureLink™ Genomic DNA Mini Kit (Invitrogen™) according to the manufacturer’s instructions. Bacterial cell lysates were generated from 10 μL of diluted *B. henselae* culture, and flea tissue lysates comprised the ground bodies of whole fleas as described above. Each gDNA extraction process utilized a negative environmental control (DNA extraction reagents without biological samples). All gDNA preparations were eluted in 50 μL of the PureLink™ genomic elution buffer and were stored in a −20 °C freezer until use. Real-time qPCR assays were performed to quantify *Bartonella* gene copy numbers as described previously [23, 25, 26]. Briefly, serial dilutions of a plasmid containing a region of the *B. henselae* citrate synthase (*gltA*) gene were used to generate a standard curve. Each qPCR reaction included 2× PowerUp SYBR Green PCR Master Mix (Applied Biosystems™), *B. henselae gltA* gene-specific primers, DNase/RNase-free water, and either gDNA template (samples), water (negative control), or serial tenfold dilutions of the plasmid containing the *B. henselae gltA* gene (3.5 × 10^8^ to 350 copies). Additionally, for flea tissue lysates, a portion of the flea 18S rDNA gene was amplified in each assay as a control to verify the integrity of the template DNA [27]. The reactions were run on a QuantStudio™ 3 real-time PCR system (Applied Biosystems™), and quantities of the *Bartonella* gene copy numbers per sample were interpolated from the standard curves.

### Quantification of immune pathway gene expression by qPCR

Nine target genes from the IMD and Toll pathways were selected based on their function in *D. melanogaster* or identification in other flea studies (IMD pathway genes: *PGRP-LC*, *IMD*, *Relish*, *attacin*, *defensin*, and *PGRP-LB*; Toll pathway genes: *PGRP-SA*, *Toll*, and *cactus*) [14, 19–21, 28]. Primer sets were generated for the cat flea from predicted mRNA sequences on the basis of the NCBI annotation of the *C. felis* transcriptome. Additionally, as previously described, annotated protein sequences from *D. melanogaster* were used as queries in BLAST searches against the *C. felis* transcriptome and *C. felis* genome to locate target genes for primer synthesis [22]. Each *C. felis* gene sequence was examined for potential RNA splice sites, and primer pairs were designed to amplify transcripts spanning a possible intron. All primer sequences generated in this study are listed in Additional file 1: Table S1.

Real-time qPCR assays were conducted to quantify the relative mRNA levels of immune pathway genes as described previously [22]. Approximately 40 flea guts were hand-dissected, pooled, and homogenized in Trizol™ reagent (InvitrogenTM) for each experimental treatment group. Total RNA was isolated using the Direct-zol™ RNA Miniprep Plus Kit (Zymo Research). The concentration of RNA was measured using a NanoDrop 2000 spectrophotometer (Thermo Fisher Scientific™), and 0.5 μg of RNA was used for complementary DNA (cDNA) synthesis using the High-Capacity RNA-to-cDNA™ Kit (Applied Biosystems™). Each qPCR reaction used 2× PowerUp SYBR Green PCR Master Mix (Applied Biosystems™), gene-specific primers, DNase/RNase-free water, and one of the following: cDNA template (samples), water (negative control), no reverse transcriptase (RT) control, or twofold serial dilutions of stock cDNA (untreated flea samples), to generate an eight-point standard curve for each gene. The reactions were run on a QuantStudio™ 3 real-time PCR system (Applied Biosystems™) using default thermal cycling conditions specified in the master mix user guide. The previously validated genes *GAPDH* and *RPL19* were used as endogenous controls [29]. Quantities of the target genes and endogenous controls were interpolated from the standard curves, and relative mRNA levels in each sample are presented as the quantity of the target gene divided by the geometric mean of the endogenous controls. Three independent trials were conducted for all experimental treatment groups.

### Quantification of antibacterial activity in vitro

Antibacterial activity was assessed in vitro using a modified assay described by Vieira et al. [30]. Using the same experimental treatment groups outlined above, approximately 40 flea guts were hand-dissected, pooled, and homogenized in PBS containing 100 μg/mL protease inhibitor (Sigma-Aldrich, catalog no. P2714-1BTL). The homogenate was centrifuged (14,000 × *g* at 4 °C for 5 min), and the supernatant was sequentially passed through two syringe membrane filters in order of decreasing pore size (0.45 μM and 0.22 μM, respectively). The filtered supernatant was collected into a new microcentrifuge tube, and samples were stored at −20 °C until use. Protein concentrations of each flea sample were measured using the Pierce™ BCA protein assay kit (Thermo Fisher Scientific™) with bovine serum albumin standards according to the manufacturer’s instructions. Antibacterial assay reactions were premixed in a microcentrifuge tube containing 30 μL of flea sample (1/4 dilution), 15 μL of Gram-positive *M. luteus* (OD_600_ = 5), 7.5 μL nutrient broth, and 66 μL of PBS. Additionally, PBS was used instead of flea samples as a positive control. The mixtures were then aliquoted in triplicate 37 μL reactions into half-area 96-well plates and were incubated overnight in a shaking incubator at 25 °C and 350 rpm. After 24 h, the total volume of each well was serially diluted, plated on nutrient agar, and incubated at room temperature (25 °C) for 5 days. The relative strength of antibacterial activity was determined by counting the resulting colonies of *M. luteus*. Also, because blood feeding alone has been shown to alter the expression of genes with an immune function [28], an additional antibacterial assay was conducted with guts collected from blood-fed, untreated control fleas at 2, 5, 7, and 14 days post-emergence to determine whether feeding-induced antibacterial activity differs with age. Three independent trials were conducted for both the previously defined experimental treatment groups and newly described age groups.

### Statistical analysis

All statistical analyses were performed using GraphPad Prism version 8 (GraphPad Software, La Jolla, CA, USA), and differences were considered significant at *p* ≤ 0.05. To analyze the expression of immune pathway genes, combined data from three independent trials were separated by time point (4 h and 24 h), and analysis of variance (ANOVA) was used to determine differences among the four experimental treatment groups (untreated control, *B. henselae*, *S. marcescens*, and *M. luteus*). Because the level of pathogenicity may differ between bacterial species, Dunnett’s multiple comparison test was used following the ANOVA to analyze differences compared with the control group only. Thus, if the *p*-value from the ANOVA revealed significant differences among the four experimental treatment groups but Dunnett’s revealed no significant differences between an infection group and the control, treatment was considered not to have a meaningful effect. The same statistical analyses were applied to examine the relative strength of antibacterial activity in vitro, except for the flea age groups. In this latter case, data were analyzed using ANOVA and Tukey’s multiple comparison test to compare differences among the four age groups (2, 5, 7, and 14 days post-emergence).

## Results

### Prevalence and intensity of bacterial infections

To examine the infection dynamics of each bacterial species within our flea system, a subset of individuals (*n* = 5) was processed after 4 h and 24 h of exposure to blood infected with either Gram-negative *B. henselae*, Gram-negative *S. marcescens*, or Gram-positive *M. luteus*. At 4 h of exposure, the infection prevalence (proportion of fleas infected with bacteria) was 67%, 70%, and 70% for *B. henselae*, *S. marcescens*, and *M. luteus*, respectively. Similarly, at 24 h of exposure, the infection prevalence was 57%, 97%, and 63% for each bacterial species, respectively. For *B. henselae*, infection intensity (mean number of *Bartonella gltA* genes per infected flea) was 2.59 × 10^5^ (± 9.95 × 10^4^ SEM) gene copies for 4 h of exposure and 1.22 × 10^4^ (± 1,678 SEM) gene copies for 24 h of exposure. For *S. marcescens* and *M. luteus*, infection intensity (mean number of CFUs per infected flea) was 998 (± 556 SEM) CFUs and 255 (± 72 SEM) CFUs at 4 h of exposure, respectively; and 872 CFUs (± 233 SEM) and 1,235 CFUs (± 486 SEM) at 24 h of exposure, respectively. Complete descriptive statistics of the infection intensity from cat fleas are listed in Additional file 2: Table S3.

### Gene expression analysis of immune pathway transcripts

**Fig. 1:**
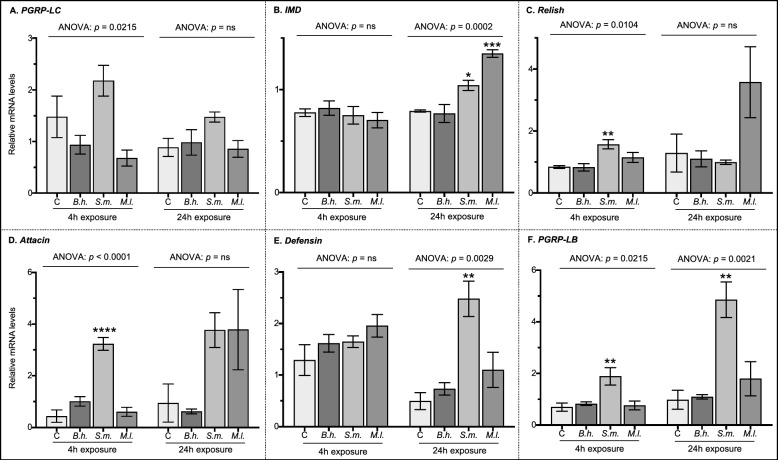
Relative mRNA levels of genes involved in the IMD pathway. The expression of **A** *PGRP-LC*, **B** *IMD*, **C** *Relish*, **D** *attacin*, **E** *defensin*, and **F** *PGRP-LB* was measured by qPCR in pooled flea samples (*n* = 40) from each of the four treatment groups (*C* control sterile blood, *B.h.* blood infected with *Bartonella henselae*, *S.m.* blood infected with *Serratia marcescens*, *M.l.* blood infected with *Micrococcus luteus*) at two exposure time points (4 and 24 h). Data are shown as the mean (± SEM) relative mRNA levels from three independent trials combined. The data were analyzed by ANOVA followed by Dunnett’s multiple comparison test in GraphPad Prism V8. Significant differences in relative mRNA levels are depicted as they relate to the control group only. Asterisks denote significance: *p ≤ 0.05, **p ≤ 0.01, ***p ≤ 0.001, ****p ≤ 0.0001. ns, not significant

**Fig. 2:**
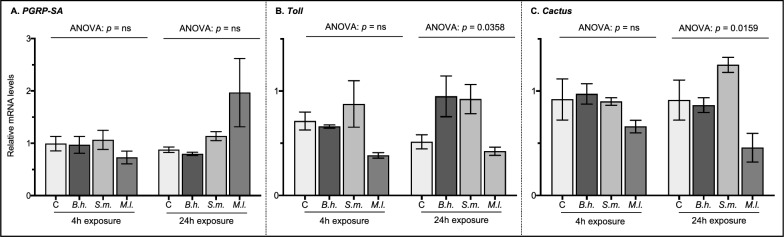
Relative mRNA levels of genes involved in the Toll pathway. The expression of **A** *PGRP-SA*, **B** *Toll*, and **C** *cactus* was measured by qPCR in pooled flea samples (*n* = 40) from each of the four treatment groups (*C * control sterile blood, *B.h.* blood infected with *Bartonella henselae*, *S.m.*  blood infected with *Serratia marcescens*, *M.l.* blood infected with *Micrococcus luteus*) at two exposure time points (4 and 24 h). Data are shown as the mean (± SEM) relative mRNA levels from 3 independent trials combined. The data were analyzed by ANOVA followed by Dunnett’s multiple comparison test in GraphPad Prism V8. Significant differences in relative mRNA levels are depicted as they relate to the control group only. ns, not significant

To examine the expression of immune pathway genes in the flea digestive tract, mRNA levels were measured by qPCR at 4 h and 24 h of exposure to either untreated control blood or blood infected with Gram-negative *B. henselae*, Gram-negative *S. marcescens*, or Gram-positive *M. luteus.* Several components of the IMD and Toll pathways were surveyed by measuring the transcription of nine genes representing three categories of pathway function: (1) activation molecules (IMD pathway: *PGRP-LC*, *IMD*, *Relish*; Toll pathway: *PGRP-SA*, *Toll*), (2) effector molecules (IMD pathway: *attacin*, *defensin*), and (3) regulatory molecules (IMD pathway: *PGRP-LB*; Toll pathway: *cactus*). At 4 h of exposure, differences in relative mRNA levels of molecules involved in the IMD pathway were only observed in *S. marcescens* treated fleas (Fig. 1). Specifically, *Relish*, *attacin*, and *PGRP-LB* were 1.9-, 7.5-, and 2.7-fold higher in this treatment group than untreated control fleas (Fig. 1C, Dunnett’s: *p* = 0.0095; Fig. 1D, Dunnett’s: *p* < 0.0001; Fig. 1F, Dunnett’s: *p* = 0.0095, respectively). Transcript levels of *PGRP-LC*, *IMD*, and *defensin* were not affected by exposure to *S. marcescens* at this time point. Again, *B. henselae* and *M. luteus* treatments did not affect mRNA levels of the IMD pathway genes at 4 h of exposure (Fig. 1). Likewise, none of the treatments affected the relative mRNA levels of molecules involved in the Toll pathway at this time point (Fig. 2).

At 24 h of exposure, differences in relative mRNA levels of molecules involved in the IMD pathway were observed in both *S. marcescens* and *M. luteus* treated fleas (Fig. 1). Specifically, *IMD* was 1.3- and 1.7-fold higher in fleas exposed to *S. marcescens* and *M. luteus*, respectively, compared with untreated control fleas (Fig. 1B, Dunnett’s: *p* = 0.0263 and *p* = 0.0002, respectively). Both *defensin* and *PGRP-LB* were 5.0-fold higher in fleas exposed to *S. marcescens* than untreated control fleas (Fig. 1E, Dunnett’s: *p* = 0.0018; Fig. 1F, Dunnett’s: *p* = 0.0018). Transcript levels of *PGRP-LC*, *Relish*, and *attacin* were unaffected by either *S. marcescens* or *M. luteus* treatments. As above, *B. henselae* did not affect mRNA levels in the IMD pathway at 24 h of exposure (Fig. 1), and none of the treatments affected the relative mRNA levels of molecules involved in the Toll pathway at this time point (Fig. 2). Overall, these data show three distinct results for each species of bacteria: (1) Gram-negative *B. henselae* (a flea-borne pathogen) does not stimulate transcriptional induction of the genes examined here for either immune signaling pathway, (2) Gram-negative *S. marcescens* (a model laboratory species) increases expression of numerous genes (*IMD*, *Relish*, *attacin*, *defensin*, and *PGRP-LB*) in the IMD pathway only, and (3) Gram-positive *M. luteus* (a model laboratory species) induces expression of an IMD-specific target gene (*IMD*) but does not stimulate transcriptional induction of the Toll pathway.

### Relative strength of antibacterial activity

**Fig. 3:**
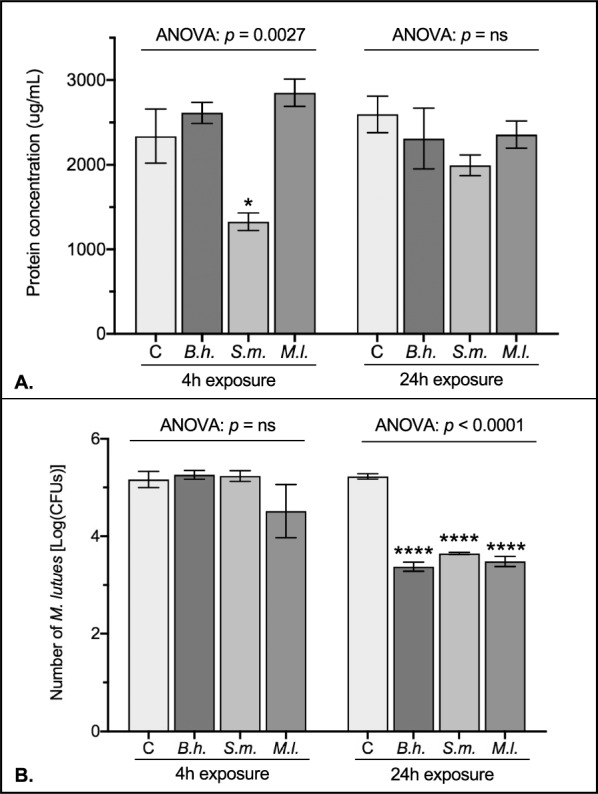
Antimicrobial activity of proteins isolated from the digestive tracts of infected fleas. Data are shown from pooled flea samples (*n* = 40) for each of the four treatment groups (*C* control sterile blood, *B.h.* blood infected with *Bartonella henselae*, *S.m.* blood infected with *Serratia marcescens*, *M.l.* blood infected with *Micrococcus luteus*) at the two exposure time points (4 h and 24 h). Column heights represent the average amount of protein in pooled flea samples (**A**) or the mean number of *M. luteus* CFUs from the antibacterial activity assay (**B**) from three independent trials. Bars represent the standard error of the mean (SEM), and data were analyzed by ANOVA followed by Dunnett’s multiple comparison test in GraphPad Prism V8. Significant differences are depicted as they relate to the control group (**C**) only. Asterisks denote significance: * *p* ≤ 0.05, ** *p* ≤ 0.01, *** *p* ≤ 0.001, **** *p* ≤ 0.0001. ns, not significant

The antibacterial activity of molecules isolated from the flea digestive tract was measured in vitro to complement the gene expression analysis of immune pathway transcripts. Because the generation of AMPs is the primary effector function of these pathways, the protein concentration of each flea sample was measured before its use in antibacterial assays with *M. luteus*. Apart from the *S. marcescens* treatment at 4 h—which was 43% lower than untreated control fleas (Fig. 3A, Dunnett’s: *p* = 0.0160)—there were no significant differences in protein concentrations between experimental treatment groups at 4 and 24 h (Fig. 3). Interestingly, despite the lower protein concentration for *S. marcescens* treatment at 4 h, there were no differences in antibacterial activity between untreated control fleas and those that were exposed to an infected bloodmeal for 4 h (Fig. 3B). In contrast, antimicrobial activity significantly increased in the digestive tracts of fleas exposed to an infected bloodmeal for 24 h compared with untreated control fleas (Fig. 3B, ANOVA: *p* < 0.0001). Specifically, molecules isolated from fleas exposed to *S. marcescens* and *M. luteus* for 24 h decreased the number of assay bacteria by 97% and 98% compared with those collected from untreated control fleas, respectively (Fig. 3B, Dunnett’s: *p* < 0.0001 and *p* < 0.0001, respectively). Similarly, the digestive tract of fleas exposed to *B. henselae* for 24 h harbored molecules that decreased the number of *M. luteus* by 99% compared with untreated control fleas (Fig. 3B, Dunnett’s: p < 0.0001). Overall, these data show that protein concentrations are comparable among cohorts of experimental treatment fleas regardless of infection history, but exposure to infected blood ultimately increases the production of antibacterial molecules in the flea digestive tract in a time-dependent manner.

**Fig. 4:**
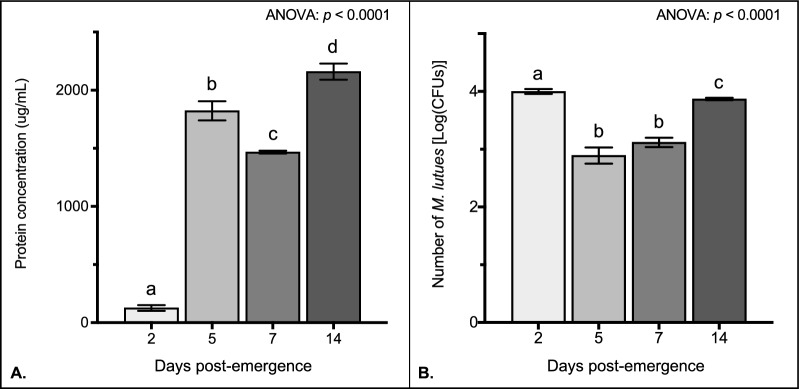
Antimicrobial activity of proteins isolated from the digestive tracts of blood-feeding fleas on different days post-emergence. Data are shown from pooled flea samples (*n* = 40) at 2, 5, 7, and 14 days post-emergence. Column heights represent the average amount of protein in pooled flea samples (**A**) or the mean number of *M. luteus* CFUs from the antibacterial activity assay (**B**) from three independent trials. Bars represent the standard error of the mean (SEM), and data were analyzed by ANOVA followed by Tukey’s multiple comparison test in GraphPad Prism V8. Different lowercase letters denote significant differences (*p* ≤ 0.05) between days. The same lowercase letters denote no significant differences between days

The protein concentration in the flea digestive tract differs significantly with age (Fig. 4A, ANOVA: *p* < 0.0001). Specifically, protein levels increased by 1347% between 2 and 5 days post-emergence (Fig. 4A, Tukey’s: *p* < 0.0001), decreased by 19% between 5 and 7 days post-emergence (Fig. 4A, Tukey’s: *p* = 0.0085), and increased by 47% between 7 and 14 days post-emergence (Fig. 4A, Tukey’s: *p* = 0.0001). Similarly, the antibacterial activity of molecules isolated from the flea digestive tract also differed significantly with age (Fig. 4B, ANOVA: *p* < 0.0001). The number of *M. luteus* CFUs was highest in 2-day-old fleas, indicating that the antibacterial activity of flea molecules was lowest at this age. The number of *M. luteus* CFUs decreased by 91% between 2- and 5-day-old fleas (Fig. 4B, Tukey’s: *p* < 0.0001), were similar between 5- and 7-day-old fleas (Fig. 4B, Tukey’s: *p* = 0.8252), and increased by 446% between 7- and 14-day-old fleas (Fig. 4B, Tukey’s: *p* < 0.0001). Together, these data suggest that protein concentrations in the digestive tract of blood-feeding fleas differ with age, corresponding to differential patterns of antibacterial activity.

## Discussion

The importance of AMPs in decreasing pathogens and curbing infection has been demonstrated in numerous blood-feeding insects, including bed bugs, lice, mosquitoes, sand flies, triatomines, and the Oriental rat flea (*Xenopsylla cheopis*) [14, 30–36]. In contrast, relatively little is known about the immune signaling pathways that control AMP gene expression in the digestive tract of cat fleas, nor the subsequent role of these peptides in the defense against ingested bacterial pathogens. The current study provides evidence in *C. felis* that (1) the upregulation of AMP transcripts coincides with the activation of the IMD pathway, (2) different species of bacteria elicit distinct transcriptional profiles of the IMD pathway genes, and (3) antibacterial molecules are produced as part of local defense mechanisms in the gut, with differential patterns of antibacterial activity related to infection status and age.

### Transcriptional response of the IMD pathway in the cat flea digestive tract

Genetic analyses in the well-studied fruit fly (*D. melanogaster*) have shown that the major receptor acting upstream of the IMD pathway is the transmembrane protein PGRP-LC, which detects peptidoglycan released from Gram-negative bacteria. Others have shown that the function of PGRP-LC is generally conserved between *Drosophila* and hematophagous arthropods (reviewed in Ref. [37]), although evidence suggests that it also responds to Gram-positive bacteria in mosquitoes [38]. Transcripts of PGRPs are expressed in insect tissues known to play a role in the immune response (e.g., hemocytes, fat body, and midgut); however, their upregulation in response to bacterial infection is variable, with many long *PGRP* (*PGRP-L*) genes constitutively expressed in certain immune tissues [39]. For example, a recent transcriptome study showed that the *PGRP-LC* gene was expressed at similar levels in both sterile blood-fed and *Y. pestis*-infected rat fleas after 4 h compared with unfed controls [14]. Similarly, in the present study, mRNA levels of *PGRP-LC* were consistent among all experimental treatment groups at each time point, all of which involved blood feeding. Together, these results may indicate that *PGRP-L* genes, such as *PGRP-LC*, are constitutively expressed in the gut of actively feeding fleas to screen ingested blood for potential pathogens.

Next, in the IMD signaling cascade, PGRP-LC recruits the adaptor protein IMD, which associates with FADD and Dredd to initiate the cleavage and subsequent translocation of the transcription factor Relish into the cell nucleus. Transduction of intracellular innate immune signaling occurs within minutes and largely depends on post-translation modifications of these proteins [40]. Nevertheless, several species of insects have shown increased transcription of *IMD* or *Relish* in the first few hours after a bacterial challenge [41–44]. The role of IMD and Relish has been demonstrated in the *C. felis*–*R. typhi* infection model, wherein the knockdown of either protein-coding gene using RNAi technology significantly increased the rickettsial burden in the flea digestive tract [21]. Likewise, transcription of *Relish* increased in rat fleas after feeding on *Y. pestis*-infected blood for 4 h [14]. Here, we observed distinct transcriptional patterns for *IMD* and *Relish* depending on the type of bacteria and the length of time fleas were exposed to an infected bloodmeal. For Gram-negative *S. marcescens*, such as *Y. pestis* infection in rat fleas, *Relish* mRNA levels increased in cat fleas after feeding on the treated blood for 4 h; however, after 24 h, *Relish* transcripts levels returned to baseline, and transcription of the *IMD* gene increased compared with control fleas. For Gram-positive *M. luteus*, neither transcript levels differed from the control group at 4 h of exposure, but, as with *S. marcescens* infection, *IMD* mRNA levels increased in *C. felis* after feeding on *M. luteus*-infected blood for 24 h. The implications of these results are discussed below in relation to AMP transcript abundance.

The translocation of the transcription factor Relish into the cell nucleus initiates the transcription of AMPs needed for pathogen clearance. The quantities and types of AMPs in insects vary significantly between species, but given their broad-spectrum bactericidal effects, defensins and attacins are found in most insects (reviewed in Ref. [4]). At least four classes of AMPs have been identified from the order Siphonaptera: attacin, cecropin, coleoptericin, and defensin [14, 19, 20, 45]. In the rat flea digestive tract, the most highly upregulated transcripts in the early response to *Y. pestis* infection (4 h post-feeding) encode for two attacins and a coleoptericin-like peptide [14]. In cat fleas, *defensin* is the more commonly detected AMP transcript, with differences in expression observed between tissues (salivary glands versus midgut) and species of *Rickettsia* (*R. felis* versus *R. typhi*) [19, 20]. Pertinent to this study, Dreher-Lesnik et al. [19] observed equal levels of *defensin* expression in the midgut of cat fleas exposed to both sterile and *R. typhi*-infected blood over the course of four days. Here, we observed an increase in transcription of two cat flea AMPs (*attacin* and *defensin*) in response to the Gram-negative bacterium *S. marcescens* only, each of which exhibited temporal patterns of expression. Again, like *Y. pestis* infection in rat fleas, *attacin* mRNA levels increased in cat fleas after feeding on *S. marcescens*-infected blood for 4 h, which coincides with the upregulation of *Relish* transcripts at this same time point. However, after 24 h, the difference in *attacin* transcript levels from control fleas was not meaningful. Instead, *defensin* mRNA levels increased, alongside the increased transcription of the adaptor protein *IMD*, but with no expression correlation to the transcription factor *Relish*. The rate of translation and protein degradation of these molecules is unknown in cat fleas, and the current study was not designed to discern the interplay between transcription factors and the genes they regulate; still, these results emphasize the potential for temporal patterns in AMP gene expression corresponding to early and late stages of infection. Moreover, like other insects, the expression of multiple AMP genes suggests that these peptides may work together to eliminate invading pathogens in the gut of cat fleas [46, 47]. In contrast to *S. marcescens*-infected fleas, no differences in AMP transcript levels were observed for fleas exposed to Gram-positive *M. luteus* despite increased transcription of the adaptor protein IMD at 24 h. Thus, like fruit flies, the IMD pathway in cat fleas may play a role in the clearance of ingested Gram-positive bacteria, but with mechanism(s) independent of AMP gene expression [10].

Finally, because constitutive immune activation is harmful to the host, the IMD pathway is modulated by negative feedback loops to ensure properly timed and adjusted immune responses. As noted above, peptidoglycan binding by the transmembrane protein PGRP-LC activates this pathway; thus, the degradation of peptidoglycan into nonimmunostimulatory fragments is generally the first level of negative regulation. In *Drosophila*, this is achieved through the secretion of catalytic peptidoglycan recognition proteins such as PGRP-LB. In contrast to other PGRP-L genes, which are largely known to be constitutively expressed, the *PGRP-LB* gene is highly inducible in response to bacterial infection [39]. In prior studies involving cat and rat fleas, it was observed that the gene *PGRP-LB* is expressed in the gut epithelium following a bloodmeal; however, the gene displayed no variation in expression levels in response to infection [14, 19, 28]. Here, we observed increased transcription of *PRGP-LB* only in fleas exposed to the Gram-negative bacterium *S. marcescens*. Moreover, expression of the *PGRP-LB* gene increased by 158% between 4 h and 24 h of exposure to the treatment blood; however, this may be indicative of the higher infection prevalence observed for *S. marcescens* at 24 h as the data collected were unpaired. Overall, our findings provide further support that the IMD pathway typifies the flea immune response to bacterial infection, but further study is needed to determine the function of these putative immune genes in *C. felis*.

### Transcriptional response of the Toll pathway in the cat flea digestive tract

The Toll pathway is another major signaling cascade in the innate immune response of insects. It is indirectly activated when the extracellular recognition protein PGRP-SA binds to peptidoglycan on the surface of Gram-positive bacteria [48–50]. This triggers a serine protease cascade that converts the extracellular cytokine Spätzle into its active form. The active Spätzle can then bind to the transmembrane receptor protein Toll. Once bound, Toll signaling directs the phosphorylation and degradation of Cactus, a regulatory protein that inhibits the NF-κB transcription factors Dif and Dorsal. Activated Dif and/or Dorsal translocate into the nucleus to initiate transcription of select insect AMP genes (*bomanin*, *drosomycin*, and *metchnikowin*). In *Drosophila*, current evidence does not suggest that the Toll signaling pathway contributes to the fight against invading microorganisms at the barrier epithelia. Instead, bacterial recognition by PGRP-SA and downstream signaling of the Toll pathway has been shown to sustain commensal bacteria in the gut of fruit flies [51]. In the digestive tract of rat fleas, transcription of the negative suppressor *Cactus* was upregulated after 4 h in both sterile blood-fed and *Y. pestis*-infected *X. cheopis* compared with unfed controls [14]. Here, in *C. felis*, mRNA levels for the three representative Toll pathway genes (*PGRP-SA*, *Toll*, and *Cactus*) were similar among all experimental treatment groups at each time point. Thus, like in other insects, the data suggest that the Toll pathway in cat fleas may play a limited role, if any, in the local immune response against ingested bacterial pathogens. Ultimately, further study is required to determine the Toll pathway’s function in the digestive tract of *C. felis*.

### Antibacterial activity of the molecules isolated from the cat flea digestive tract

Numerous studies have shown the complex connection between the transcription of immune genes and the resulting antibacterial activity in insects [34]. Antibacterial activity ultimately occurs from the production of humoral defense molecules that prevent bacterial growth; consequently, it can be influenced by factors unrelated to gene expression. In the present study, despite the increase in levels of *attacin* mRNA in *S. marcescens*-infected fleas, the antibacterial activity of molecules isolated from flea digestive tracts was similar across all experimental treatment groups after 4 h. However, after 24 h, the molecules isolated from fleas fed an infected bloodmeal (*B. henselae*, *S. marcescens*, or *M. luteus*) demonstrated potent antibacterial activity compared with blood-fed control fleas. Apart from the upregulation of *defensin* transcription in *S. marcescens*-infected fleas, the increase in antibacterial activity at 24 h appears mostly independent of the observed gene expression pattern. Several factors could be responsible for this discrepancy in cat fleas. First, we examined a select few genes from the IMD and Toll pathways based primarily on their function in *D. melanogaster*. Although most insects share the same basic architecture of the immune system as fruit flies, there is a great deal of diversity in their immune components. Thus, a *C. felis* transcriptome study utilizing RNA sequencing may reveal other immune genes related to the increased antibacterial activity. Secondly, the timing and duration of immune gene expression can vary widely, with many genes being transiently expressed within the first 24 h after infection [52]. Although we examined two distinct time points after infection in *C. felis*, a comprehensive time-course analysis between 4 h and 24 h may reveal temporal changes in gene expression levels that were missed in the current study. Finally, the insect intestinal tract utilizes the local production of ROS and AMPs to establish two complementary defense mechanisms. In fleas, it has been shown that ROS synthesis in the gut plays an important role in the immune response [22, 53]. However, there is often limited expression correlation of genes related to ROS generation, which has raised questions about the role of ROS production in the initial flea immune response to infection [14, 22]. Thus, the molecules isolated from the digestive tract of cat fleas may encompass other antimicrobial factors not examined here. Together, these data highlight the need to supplement gene expression analysis with the subsequent production of antibacterial molecules to gain a comprehensive understanding of the flea immune response.

In most animals, the efficiency of the immune system decreases with age. This can have significant implications for the spread of vector-borne pathogens as aging insects may become more susceptible to infection. Here, we showed that the antibacterial activity of molecules isolated from the digestive tract of blood-feeding cat fleas exhibits a slightly bimodal distribution with age. Specifically, during the 2 weeks following emergence, the antibacterial activity of molecules produced in response to blood feeding alone was minimal at 2 days, peaked at 5 days and 7 days, and then decreased to a moderate level at 14 days. In contrast to other blood-feeding insects that feed relatively infrequently by consuming a single bloodmeal and digesting it over several days (e.g., mosquitoes, kissing bugs, and sandflies), cat fleas remain close to their host and may feed multiple times per day [54]. Additionally, although generation times are relatively short (about 1 month), adult fleas can survive for up to 100 days or more on their host [55]. Thus, these findings highlight the importance of investigating age-related changes in *C. felis* immunity, which can ultimately influence pathogen transmission.

### Cat flea immunity against the flea-borne pathogen* Bartonella henselae*

Few studies have been done on how the flea immune system responds to flea-borne pathogens [14, 19–21, 53, 56]. Moreover, apart from *Y. pestis*, little is known about how these pathogens behave inside their flea hosts. *Bartonella* species can survive in their vertebrate hosts by evading the immune system (reviewed in [57]). It is hypothesized that they do this by changing their surface proteins to circumvent immune recognition and by having diverse lipopolysaccharide structures that help them avoid detection by pattern recognition receptors [58–60]. In the current study, infection with *B. henselae* did not stimulate transcriptional induction of any flea immune pathway genes examined. However, the molecules isolated from the digestive tract of *B. henselae*-infected fleas showed significantly higher antibacterial activity than control fleas, which suggests activation of the flea immune system. Similar results have been reported from the human body louse (*Pediculus humanus humanus*) infected with a related vector-borne pathogen, *Bartonella quintana*. Specifically, transcript levels of peptidoglycan recognition proteins and defensins did not differ between control and *B. quintana*-infected lice [61]. Yet, when the antibacterial activity of recombinant louse defensins was tested in vitro, these molecules were found to limit the growth of *B. quintana* [62]. Additional research is essential to better understand how the flea immune system responds to *Bartonella* infection. Furthermore, it is important to investigate how *B. henselae* evades these immune responses to ensure its survival and proliferation within the flea host.

Finally, contrary to *B. henselae*, bacterial infection with the two non-flea-borne pathogens (*S. marcescens* and *M. luteus*) stimulated the transcriptional induction of several flea immune pathway genes examined. A couple of factors could be responsible for this observed difference. First, as reviewed above, stimulation of both the IMD and Toll pathways occurs via recognition of peptidoglycan shed during bacterial replication. *Bartonella* species are slow-growing organisms with an average dividing time of approximately 24 h, whereas *S. marcescens* and *M. luteus* cells may replicate about every hour [63–65]. Thus, the increased transcription of flea immune pathway genes may correspond to the quantity of peptidoglycan released from these faster-dividing bacteria. Second, the bacterial species in this study may differ in their capacity to cause harm to the flea host. For example, although not tested here, *S. marcescens* can be a lethal insect pathogen because it can invade the midgut epithelium and penetrate the body cavity, ultimately causing septicemia [66, 67]. In contrast, *B. henselae* causes almost no lethality in fleas and has only been found to cause extracellular infections in the intestinal lumen [26, 68]. Therefore, aside from shedding peptidoglycan, *S. marcescens* may also stimulate the flea immune response by generating cellular damage signals.

## Conclusions

This research significantly enhances our understanding of the intricate immune signaling pathways and antibacterial mechanisms within the digestive tract of fleas. The findings indicate that the IMD pathway serves as a primary immune response in fleas against ingested bacteria. However, it is worth noting that the flea-borne pathogen *B. henselae* might have evolved mechanisms to evade activating this specific immune signaling pathway. Despite this, foreign bacteria in the flea gut stimulate the production of antimicrobial molecules, which goes beyond the effects of blood feeding alone. In conclusion, this research presents crucial findings regarding the immune response of the cat flea vector.

## Supplementary Information

Additional file 1: Table S1. Information on the primers used in this study.

Additional file 2: Table S2. Descriptive statistics of the infectious dose of infected bloodmeals. The infectious dose of *Bartonella henselae* was determined by quantifying *Bartonella* gene copy numbers using qPCR and genomic DNA extracted from the bacterial culture. The infectious doses for *Serratia marcescens* and *Micrococcus luteus* were determined by plating serial dilutions of the bacterial cultures on nutrient agar and counting the resultant colony forming units (CFUs). Data are shown from six independent trials combined. h,  hours of exposure; SEM, standard error of the mean. Table S3. Descriptive statistics of bacterial load from infected cat fleas. The amount of *Bartonella henselae* was determined by measuring *Bartonella* gene copy numbers using qPCR and genomic DNA extracted from individual flea samples. The quantities of *Serratia marcescens* and *Micrococcus luteus* were determined by plating a diluted sample of each flea on nutrient agar and counting the resultant colony forming units (CFUs). Data are shown from six combined independent trials, except *B. henselae* data at 4 h of exposure, which are shown from three independent trials (*n* = no. of positive fleas/total no. of fleas tested). Data does not include zero values from uninfected fleas. h, hours of exposure; SEM, standard error of the mean.

## Data Availability

The data supporting the conclusions of this manuscript are included within the article.

## Abbreviations

cDNA: Complementary deoxyribonucleic acid
CFUs: Colony forming units
gDNA: Genomic deoxyribonucleic acid
PBS: Phosphate-buffered saline
qPCR: Quantitative polymerase chain reaction
SEM: Standard error of the mean
TSA: Tryptic soy agar
TSB: Tryptic soy broth
