# Supplementary material for: Transcriptional induction of the IMD signaling pathway and associated antibacterial activity in the digestive tract of cat fleas (Ctenocephalides felis)
Source: Parasit Vectors. 2024 Dec 30;17:546. doi: 10.1186/s13071-024-06613-x (PMC11687193; doi:10.1186/s13071-024-06613-x)
Supplement: Supplementary file 1 — Additional file 1: Table S1. Information on the primers used in this study. [file 13071_2024_6613_MOESM1_ESM.docx]

**Additional file 2: Table S2.** Descriptive statistics of the infectious dose of infected bloodmeals. The infectious dose of *Bartonella* *henselae* was determined by quantifying *Bartonella* gene copy numbers using qPCR and genomic DNA extracted from the bacterial culture. The infectious doses for *Serratia marcescens* and *Micrococcus luteus* were determined by plating serial dilutions of the bacterial cultures on nutrient agar and counting the resultant colony forming units (CFUs). Data are shown from 6 independent trials combined. h = hours of exposure; SEM = standard error of the mean.

|  | *Bartonella henselae* (gene copies) | | *Serratia marcescens* (CFUs) | | *Micrococcus luteus* (CFUs) | |
| --- | --- | --- | --- | --- | --- | --- |
|  | 4 h | 24 h | 4 h | 24 h | 4 h | 24 h |
| Mean | 3.90E+09 | 9.96E+08 | 1.35E+08 | 3.68E+07 | 4.46E+07 | 1.85E+07 |
| Minimum | 2.21E+09 | 1.11E+08 | 2.44E+07 | 1.36E+06 | 2.97E+07 | 6.77E+05 |
| Maximum | 5.05E+09 | 2.36E+09 | 3.58E+08 | 1.50E+08 | 6.20E+07 | 5.12E+07 |
| SEM | 8.62E+08 | 4.25E+08 | 5.32E+07 | 2.33E+07 | 5.80E+06 | 8.10E+06 |

**Additional file 2. Table S3.** Descriptive statistics of bacterial load from infected cat fleas. The amount of *Bartonella henselae* was determined by measuring *Bartonella* gene copy numbers using qPCR and genomic DNA extracted from individual flea samples. The quantities of *Serratia marcescens* and *Micrococcus luteus* were determined by plating a diluted sample of each flea on nutrient agar and counting the resultant colony forming units (CFUs). Data are shown from 6 combined independent trials, except *B. henselae* data at 4 hours of exposure are shown from three independent trials (*n* = # of positive fleas/total # of fleas tested). Data does not include zero values from uninfected fleas. h = hours of exposure; SEM = standard error of the mean.

|  | *Bartonella henselae* (gene copies) | | *Serratia marcescens* (CFUs) | | *Micrococcus luteus* (CFUs) | |
| --- | --- | --- | --- | --- | --- | --- |
|  | 4 h  (*n* = 10/15) | 24 h  (*n* = 17/30) | 4 h  (*n* = 21/30) | 24 h  (*n* = 29/30) | 4 h  (*n* = 21/30) | 24 h  (*n* = 19/30) |
| Mean | 2.59E+05 | 1.22E+04 | 9.98E+02 | 8.72E+02 | 2.55E+02 | 1.24E+03 |
| Minimum | 5.38E+04 | 5.38E+03 | 7.00E+00 | 7.00E+00 | 7.00E+00 | 7.00E+00 |
| Maximum | 1.10E+06 | 2.70E+04 | 8.71E+03 | 5.48E+03 | 1.22E+03 | 6.28E+03 |
| SEM | 9.95E+04 | 1.68E+03 | 5.56E+02 | 2.33E+02 | 7.20E+01 | 4.86E+02 |
