# Supplementary material for: Transcriptional induction of the IMD signaling pathway and associated antibacterial activity in the digestive tract of cat fleas (Ctenocephalides felis)
Source: Parasit Vectors. 2024 Dec 30;17:546. doi: 10.1186/s13071-024-06613-x (PMC11687193; doi:10.1186/s13071-024-06613-x)
Supplement: Supplementary file 2 — Additional file 2: Table S2. Descriptive statistics of the infectious dose of infected bloodmeals. The infectious dose of Bartonella henselae was determined by quantifying Bartonella gene copy numbers using qPCR and genomic DNA extracted from the bacterial culture. The infectious doses for Serratia marcescens and Micrococcus luteus were determined by plating serial dilutions of the bacterial cultures on nutrient agar and counting the resultant colony forming units (CFUs). Data are shown from six independent trials combined. h, hours of exposure; SEM, standard error of the mean. Table S3. Descriptive statistics of bacterial load from infected cat fleas. The amount of Bartonella henselae was determined by measuring Bartonella gene copy numbers using qPCR and genomic DNA extracted from individual flea samples. The quantities of Serratia marcescens and Micrococcus luteus were determined by plating a diluted sample of each flea on nutrient agar and counting the resultant colony forming units (CFUs). Data are shown from six combined independent trials, except B. henselae data at 4 h of exposure, which are shown from three independent trials (n = no. of positive fleas/total no. of fleas tested). Data does not include zero values from uninfected fleas. h, hours of exposure; SEM, standard error of the mean. [file 13071_2024_6613_MOESM2_ESM.docx]

**Additional file 1: Table S1.** Information on the primers used in this study.

| Gene name (abbreviation) [target organism] | Primer sequence (5'→3') | Reference |
| --- | --- | --- |
| Peptidoglycan-recognition protein LC-like (PGRP-LC) [*C. felis*] | F: GTACTGGCGTTTTGCCTTGT | This study |
|  | R: CCACCCATTCTTGACGAGAT |  |
| Immune deficiency (IMD) [*C. felis*] | F: TGCAAAATGTCAATCGAGGA | This study |
|  | R: GGCCTAACTGGAGCATCATC |  |
| Relish (Rel) [*C. felis*] | F: TGCTTTTGGATCGTTCAGTG | This study |
|  | R: GCGATCAGAACCCAATATACG |  |
| Attacin-A-like (Attacin) [*C. felis*] | F: GCAGACCAGGCAGAAAAGAC | This study |
|  | R: CTGAAGACCTCCTCCGACAG |  |
| Defensin-like (Defensin) [*C. felis*] | F: CGGGTTAGTGTTGGTGGTTT | This study |
|  | R: GAGATATGCACCGAGCATTG |  |
| Peptidoglycan-recognition protein LB-like (PGRP-LB) [*C. felis*] | F: ATGGCTGCGATTAGGACATT | This study |
|  | R: CACCACATGCTCCAGGAATA |  |
| Peptidoglycan-recognition protein SA-like (PGRP-SA) [*C. felis*] | F: TGAAGGTGCAGGATGGTATAAA | This study |
|  | R: CAAGTGCTCCTAGTGCAACG |  |
| Protein toll-like (Toll) [*C. felis*] | F: TTGTCGAATGAACTGGTGCG | This study |
|  | R: ACATACCGTCAAACACCCCT |  |
| NF-kappa-B inhibitor cactus-like (Cactus) [*C. felis*] | F: TCAGGAGCCATCTGTCATTG | This study |
|  | R: CTTTGCACGCCTCTGTCATA |  |
| Glyceraldehyde-3-phosphate dehydrogenase (GAPDH) [*C. felis*] | F: ACCCAAAAGACTGTGGATGG | [29] |
|  | R: CGGAATGACTTTGCCTACAG |  |
| Ribosomal protein L19 (RPL-19) [*C. felis*] | F: TACAGCTAATGCCCGTACACC | [29] |
|  | R: TTCAACAAACGCCTCAGGAC |  |
| *C. felis* 18S rDNA (Cf18S) [flea] | F: TGCTCACCGTTTGACTTGG | [27] |
|  | R: GTTTCTCAGGCTCCCTCTCC |  |
| Citrate synthase (gltA) [*Bartonella* genus] | F: GTGCTAATCCATTTGCATGTATT | [26] |
|  | R: GTAACATTTTTAGGCATGCTTCATTA |  |
